# Supplementary material for: Causal Relationship between Aging and Anorexia Nervosa: A White-Matter-Microstructure-Mediated Mendelian Randomization Analysis
Source: Biomedicines. 2024 Aug 16;12(8):1874. doi: 10.3390/biomedicines12081874 (PMC11351342; doi:10.3390/biomedicines12081874)
Supplement: Supplementary file 1 [file biomedicines-12-01874-s001.zip › Supplementary Figure/Figure S4/Singe SNP plot of MR analysis for TL to ubm-b-1856.pdf]

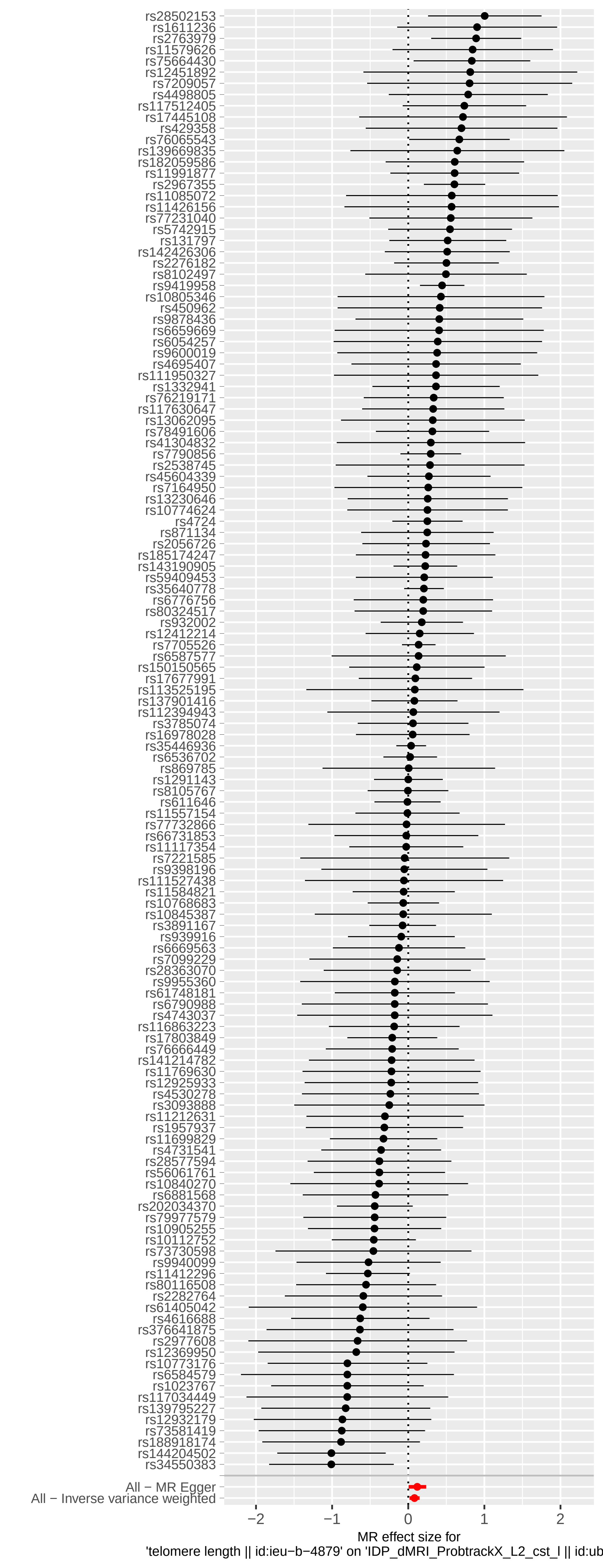
rs28502153
rs1611236
rs2763979
rs11579626
rs75664430
rs12451892
rs7209057
rs4498805
rs117512405
rs17445108
rs429358
rs76065543
rs139669835
rs182059586
rs11991877
rs2967355
rs11085072
rs11426156
rs77231040
rs5742915
rs131797
rs142426306
rs2276182
rs8102497
rs9419958
rs10805346
rs450962
rs9878436
rs6659669
rs6054257
rs9600019
rs4695407
rs111950327
rs1332941
rs76219171
rs117630647
rs13062095
rs78491606
rs41304832
rs7790856
rs2538745
rs45604339
rs7164950
rs13230646
rs10774624
rs4724
rs871134
rs2056726
rs185174247
rs143190905
rs59409453
rs35640778
rs6776756
rs80324517
rs932002
rs12412214
rs7705526
rs6587577
rs150150565
rs17677991
rs113525195
rs137901416
rs112394943
rs3785074
rs16978028
rs35446936
rs6536702
rs869785
rs1291143
rs8105767
rs611646
rs11557154
rs77732866
rs66731853
rs11117354
rs7221585
rs9398196
rs111527438
rs11584821
rs10768683
rs10845387
rs3891167
rs939916
rs6669563
rs7099229
rs28363070
rs9955360
rs61748181
rs6790988
rs4743037
rs116863223
rs17803849
rs76666449
rs141214782
rs11769630
rs12925933
rs4530278
rs3093888
rs11212631
rs1957937
rs11699829
rs4731541
rs28577594
rs56061761
rs10840270
rs6881568
rs202034370
rs79977579
rs10905255
rs10112752
rs73730598
rs9940099
rs11412296
rs80116508
rs2282764
rs61405042
rs4616688
rs376641875
rs2977608
rs12369950
rs10773176
rs6584579
rs1023767
rs117034449
rs139795227
rs12932179
rs73581419
rs188918174
rs144204502
rs34550383
All − MR Egger
All − Inverse variance weighted
−2
−1
0
1
2
MR effect size for
'telomere length || id:ieu−b−4879' on 'IDP_dMRI_ProbtrackX_L2_cst_l || id:ub
